# Nanoscale Design of Nano-Sized Particles in Shape-Memory Polymer Nanocomposites Driven by Electricity

**DOI:** 10.3390/ma6093742

**Published:** 2013-09-02

**Authors:** Haibao Lu, Wei Min Huang, Fei Liang, Kai Yu

**Affiliations:** 1 Science and Technology on Advanced Composites in Special Environments Laboratory, Harbin Institute of Technology (HIT), Harbin 150080, China; 2 School of Mechanical & Aerospace Engineering College of Engineering, Nanyang Technological University, 639798, Singapore; 3 Department of Mechanical, Materials & Aerospace Engineering, University of Central Florida, Orlando 32826, FL, USA

**Keywords:** smart materials, nanoscale design, nanocomposites, shape-memory polymers

## Abstract

In the last few years, we have witnessed significant progress in developing high performance shape memory polymer (SMP) nanocomposites, in particular, for shape recovery activated by indirect heating in the presence of electricity, magnetism, light, radio frequency, microwave and radiation, *etc.* In this paper, we critically review recent findings in Joule heating of SMP nanocomposites incorporated with nanosized conductive electromagnetic particles by means of nanoscale control via applying an electro- and/or magnetic field. A few different nanoscale design principles to form one-/two-/three- dimensional conductive networks are discussed.

## 1. Introduction

Shape-memory polymers (SMPs) not only open an exciting field for many potential applications, but also bring forward a significant breakthrough in the development of advanced stimulus-responsive materials to complement or supplant traditional materials and approaches in a variety of engineering applications [1,2,3]. SMPs are characterized by the shape-memory effect (SME), which is defined as the ability of a material to regain its original shape in the presence of the right stimulus [4]. For a heating-responsive SMP, in which the stimulus is heat, we may easily deform it at relatively high temperatures and, then, cool it down, with the deformed shape being maintained [5,6]. After removal of constraint, apart from slight elastic recovery, the free-standing material is able to largely keep the deformed shape. Elastic energy is stored in the deformed polymer chains. This is the programming process. Upon reheating for shape recovery, the polymer softens and the polymer chains release the stored elastic energy, and, consequently, shape recovery is observed if there is no constraint applied (*i.e.*, free recovery) [7,8,9]. In the case of constrained recovery, the maximum stress generated upon heating is dependent on the elastic energy stored during programming [10]. The actual shape memory performance in a particular SMP is determined by its chemical structure, molecular weight, degree of cross-linking, fraction of amorphous and crystalline domains, *etc.* [11,12,13]. In addition to direct heating, heating-responsive SMPs may be triggered via indirect heating by means of applying electricity, magnetism, light, radio frequency, microwave or radiation [14,15,16,17,18]. It is also possible to combine more than one stimulus together for simultaneous triggering or in a step-by-step manner [10,12,19,20,21]. So far, a range of SMPs have been used in many engineering applications, from aerospace engineering to biomedical engineering [3,22,23,24,25].

In the case of Joule heating, normally electrical conductive fillers are incorporated into a SMP to improve its electrical conductivity [26,27,28,29,30,31,32,33,34,35]. This electricity triggered SME is convenient to apply in many real practices and is especially useful in some applications, such as a self-deployable aerospace structure, a morphing structure, an implanted biomedical device, *etc*., where direct heating is not easily achievable. A variety of conductive fillers, such as carbon nanoparticle, nickel powder, carbon nanotubes (CNTs) [26,27], carbon nanofibers (CNFs) [28], carbon nanopaper [29] and continuous carbon fiber [30,31,32,33,34], have been used to realize good electrical conductivity for Joule heating of SMPs. However, how to effectively integrate nano-sized particles into SMPs for high performance is still an open question at this moment [35]. The actual dispersion of conductive particles is critical in order to take full advantage of the excellent properties of individual nano-sized particles. So far, the resulting electrical conductivity of SMP composites is still relatively low, largely due to the lack of effective techniques to form conductive networks, although various strategies, including filler surface treatment, *in situ* polymerization, applying high aspect ratio particles (even continuous fibers), formation of conductive metallic particle chains or aligning particles and using hybrid fillers with a synergic effect, *etc*., have been explored [13,36].

We can see clearly that it is critically important to develop a systematic approach to integrate the unique structure and excellent properties of nano-sized particles for high shape memory performance at macroscopic scale applications [37]. Recently, CNTs have been assembled into one-dimensional fibers and two-dimensional sheets. An electrical conductivity of 10^3^ S∙cm^−1^ has been achieved in composites embedded with such fibers/sheets [38,39,40,41]. The integration of assembled CNTs into a SMP matrix for actuation via Joule heating has been explored [41]. Great efforts have been devoted to design and optimize the aligned structure for improved performance of SMP nanocomposites, in particular, for high electric conductivity.

**Figure 1:**
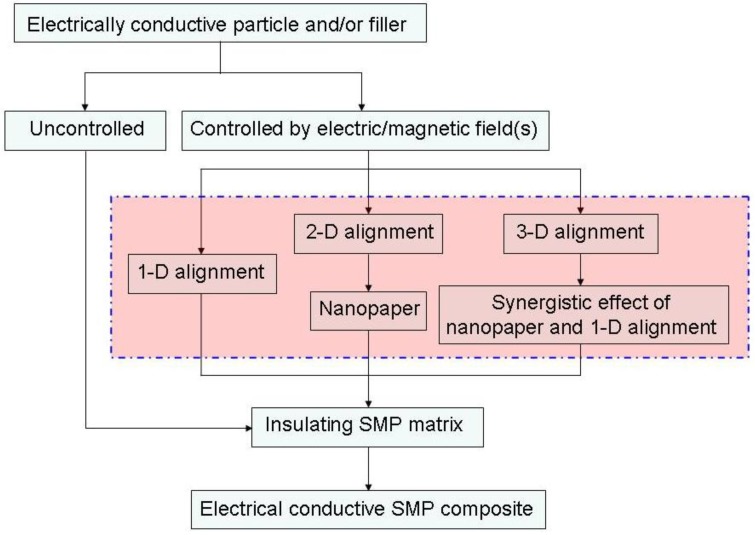
An initial diagram showing the mechanism of actuation via electrically resistive Joule heating of shape-memory polymer (SMP) nanocomposite.

This paper aims to provide an extensive review on major aspects (from design principles to engineering practice) of the nanoscale design of SMP nanocomposites with nano-sized electrically conductive particles and films (including CNTs, CNFs, nanopaper, *etc*.) for Joule heating-triggered shape recovery, together with revealing the great potentials after manipulating nano-particles into special configurations for high electrical conductivity. The most important types of techniques for controlled synthesis are reviewed, and the underlying physical mechanism is discussed in detail. This review focuses more on recent advances in the design principles of one-/two-/three-dimensional conductive networks at the nanoscale. Some issues about electrically conductive SMP nanocomposites, which have not been previously well explored, but which have great potentials, are discussed. Finally, a few typical examples are presented and a brief outlook into future aspects is added at the end of this article. An initial diagram showing the mechanism of actuation via electrically resistive Joule heating of SMP nanocomposite is presented in Figure 1.

## 2. Nanoscale Design for One-Dimensional Alignment

**Figure 2:**
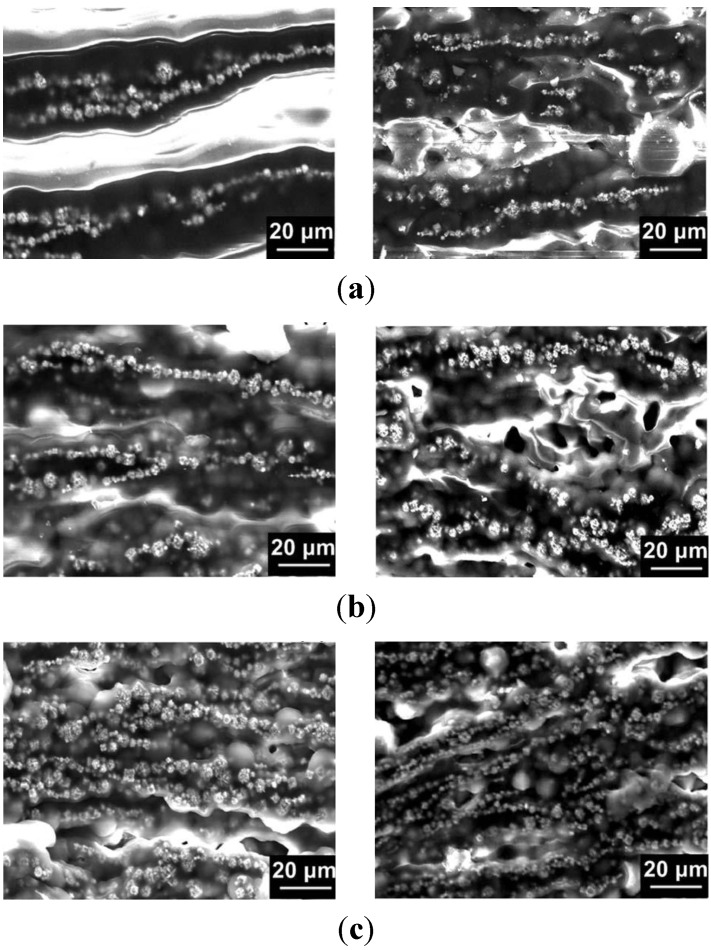
Typical scanning electron microscopy (SEM) images prior to (left column) and after (right column) five stretching-recovery cycles of the Ni chains in three SMP nanocomposites with a different volume fraction of Ni powder: (**a**) 5%; (**b**) 10%; (**c**) 20%. Reproduced with permission from [43]. Copyright 2008 American Institute of Physics.

Conductive CNTs and CNFs have been introduced into SMPs to reduce their electrical resistivity in order to be suitable for Joule heating [42,43]. On the other hand, these conductive particles also improve the thermal conductivity of the resultant SMP nanocomposites [42]. The actual configuration of these conductive channels is rather difficult to be precisely controlled, since the dispersion of these particles is determined by many factors. Ni particles, which are not only magnetic, but also electrically conductive, can be used to form conductive chains within polyurethane SMP for enhanced electrical conductivity [43]. The composite is fabricated in the following steps. First, Ni particles are blended into polyurethane, which is dissolved in dimethylformamide (DMF), and then, a magnetic field is applied. After curing for 24 h at a constant temperature of 80 °C, SMP nanocomposite with aligned Ni chains inside is obtained. Scanning electron microscopy (SEM) is used to reveal the details of the Ni chains in the SMP composite, as shown in Figure 2. As we can see, with the increase in Ni content, more Ni chains are formed, and eventually, no clear Ni chain structure can be identified. Experimental results reveal that the electrical resistivity of the composite filled with 10.0 vol % of Ni in random dispersion is 2.36 × 10^4^ Ω∙cm, while simply by forming Ni chains, it drops to 12.18 Ω∙cm. So that it can be activated by applying a voltage of 6 V for shape recovery, if carbon black is employed, by means of adding in 0.5 vol % of Ni and forming chains, the resistivity of the resultant composites can be reduced by more than 10 times [44]. The remarkable reduction in the electrical resistivity was suggested to be the result of the conductive chains, which serve as conductive channels to bridge those small isolated carbon black aggregations. Consequently, the tested SMP composite with 10 vol % carbon black and 0.5 vol % chained Ni particles can be heated to 80 °C for recovery at 1.2 W of electric power.

**Figure 3:**
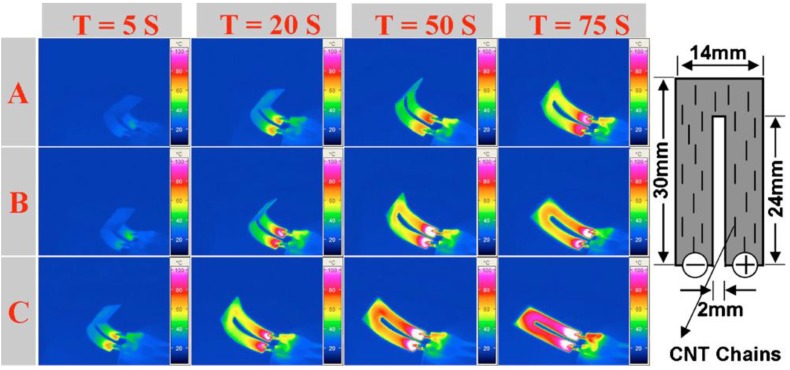
Snapshot of shape recovery process (and temperature distribution). Sample A: SMP/CB; Sample B: SMP/CB/CNT (random); Sample C: SMP/CB/CNT (chained). Right inset: sample dimension and experimental setup for Sample C. Reproduced with permission from [46].Copyright 2011 American Institute of Physics.

Instead of magnetically aligning Ni powder into chains, CNT chains have been formed by applying an electrical field before/during the curing of SMP. In the fabrication process, both carbon black and CNTs were blended into the polymer matrix, which was dissolved in DMF solvent in order to reduce the viscosity of the mixture and, thus, enhance the mobility of CNTs [45]. The distance between two electrodes was 3 cm, and the applied electrical voltage was 500 V (AC). Finally, the entire apparatus was placed in an air-tight box and kept in an oven for 24 h at 80 °C for curing. After the volatilization of the DMF solvent, SMP nanocomposite embedded with aligned CNTs was produced. The alignment of CNTs was substantiated by observation under a microscope. No large aggregates were found, indicating uniform distribution and the close packing of the alignment. The individual CNTs were initially polarized into nano-sized dipoles under the applied electric field. The attraction force dominates the local force field, and hence, CNTs attract each other to assemble into chains, rather than individually aligning along the external electric field. The reason behind this is the small distance among individual CNTs and the strong Coulombic force between oppositely charged ends. When the size of the assembled CNTs reach a critical point, the average distance among these chains increases significantly. Consequently, CNTs start to align and form chains within the SMP matrix when the aligning force induced by the external electric field is over the local Coulombic force. Experimental results prove that the electrical resistivity of the SMP nanocomposite filled with chained CNTs is reduced by more than 100 times in comparison with those only filled with randomly dispersed CNTs. As demonstrated, shape recovery in an SMP nanocomposite filled with 1.0 wt % of aligned CNTs and 15.0 wt % of carbon black can be activated by applying an electric voltage of 25 V (Figure 3 [46]). However, the conductive chains may be destroyed after several shape memory cycles.

## 3. Nanoscale Design with Two-Dimensional Nanopaper

**Figure 4:**
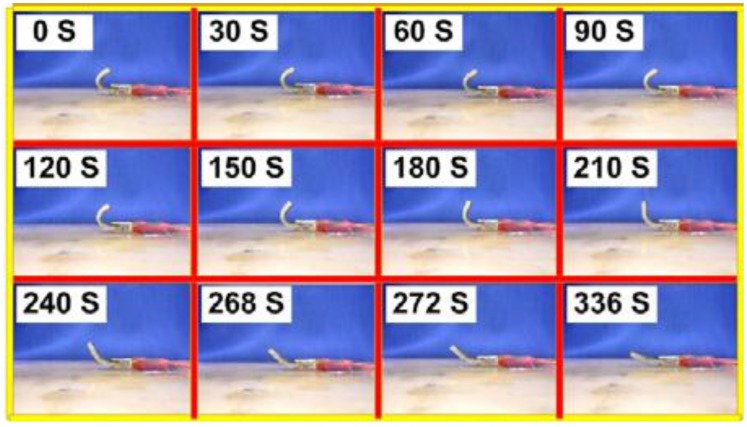
Snapshot of the shape recovery process in an SMP nanocomposite integrated with 1.2 g of CNT nanopaper. Reproduced with permission from [49]. Copyright 2012 Wiley-Vch Verlag Gmbh & Co.

Although the electrical resistivity along the direction of aligned Ni powder or CNTs is remarkably reduced, in the perpendicular direction, the electrical resistivity is still high and so is the thermal conductivity. It is easy to understand that high thermal conductivity is important for a quick reaction in thermo-responsive SMPs. Hence, two-dimensional conductive networks are preferable. Motivated by this, nanopaper made of conductive CNTs or CNFs has been scaled up to be applied in novel functional materials [47,48,49]. Consequently, the combination of conductive nanopaper and SMP is just right for electricity activated SME [50,51]. Nanopaper is an assembly of CNTs or CNFs into thin film form. The use of non-ionic surfactants helps to improve the dispersion of CNTs or CNFs in a solution [52]. Normally, nanopaper has a porous structure with individuals entangled with each other. No large aggregates are formed, which indicates a rather uniform distribution and close packing. Continuous non-woven nanotubes or nanofibers work as a continuous network to significantly improve the electrical conductivity. Shape-memory nanocomposites are fabricated by integrating nanopaper into a SMP matrix. The electrical resistivity of an SMP nanocomposite depends on the actual weight fraction in nanopaper. Due to the excellent two-dimensional network in nanopaper, both the amplitude of electrical current and the current-carrying capability increase. High efficiency of SMP nanocomposites integrated with nanopaper for Joule heating has been experimentally demonstrated. With one coated layer of CNT nanopaper of 1.2 g, an SMP nanocomposite took 336 s for full shape recover under an applied DC voltage of 7.1 V (Figure 4 [49]).

An alternative approach to make two dimensional conductive films is the electrospinning method [53]. Poly(acrylonitrile) (PAN) is used as the precursor and initially electrospun into non-woven fibers. The resulting PAN fiber mat is then converted into CNFs via a two-step process, including stabilization (or pre-oxidization) and carbonization. Fiber structure is well preserved after each step. Continuous non-woven CNFs are utilized as a conductive network for high electrical conductivity. Nanometer-sized fiber morphology also provides a percolating conductive network with a large degree of interconnectedness, which yields not only high electrical conductivity, but also high thermal conductivity, thus resulting in a higher actuation speed. Experimental results show that epoxy-based SMP nanocomposites incorporated with non-woven CNF fibers exhibit rapid electrical actuation capability. Under a constant DC voltage of 20 V, the SMP nanocomposite with a low electrical resistivity of 0.0327 Ω cm ± 0.0008 Ω cm takes 2.1 s to complete shape recovery.

In comparison with one-dimensional alignment, two-dimensional nanopaper enables SMP nanocomposites to have unprecedented high speed electrical actuation. Besides being easy for processing and having excellent electrical conductivity, two-dimensional nanopaper or non-woven CNF simultaneously enhances the thermal conductivity and electrothermal effectiveness of SMP composites.

## 4. Nanoscale Design of Three-Dimensional Network

**Figure 5:**
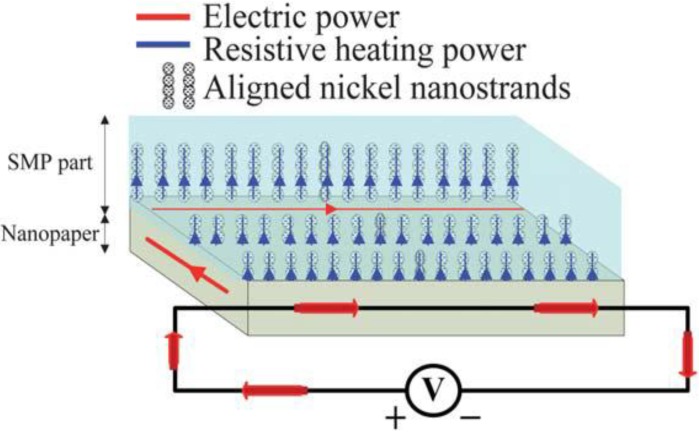
Schematic illustration of vertically aligned nickel nanostrands to help resistive heating power to transfer from the nanopaper to the underlying SMP. Reproduced with permission from [41]. Copyright 2011 RSC Publishing.

To further improve electrically-induced shape recovery in SMP nanocomposites coated with carbon nanopaper, the synergistic effect of carbon nanopaper and nickel nanostrand (or nickel coated CNT) alignments was exploited for electrical actuation of SMP nanocomposites [53,54]. The combination of carbon nanopaper and nickel nanostrand alignments was initially used to improve both the thermal and electrical conductivities of SMP to avoid the negative effect of randomly dispersed particles on the shape recovery ratio [41]. The in-plane conductive nanopaper was coated on the surface of an SMP to achieve the actuation of nanocomposites by Joule heating. Meanwhile, the nickel nanostrand alignments were embedded into the SMP matrix to improve the speed of heat transfer from the nanopaper to the SMP nanocomposite. As a result, the speed for electrically-induced shape recovery in SMP nanocomposites was significantly accelerated. An illustration is presented in Figure 5 [41].

**Figure 6:**
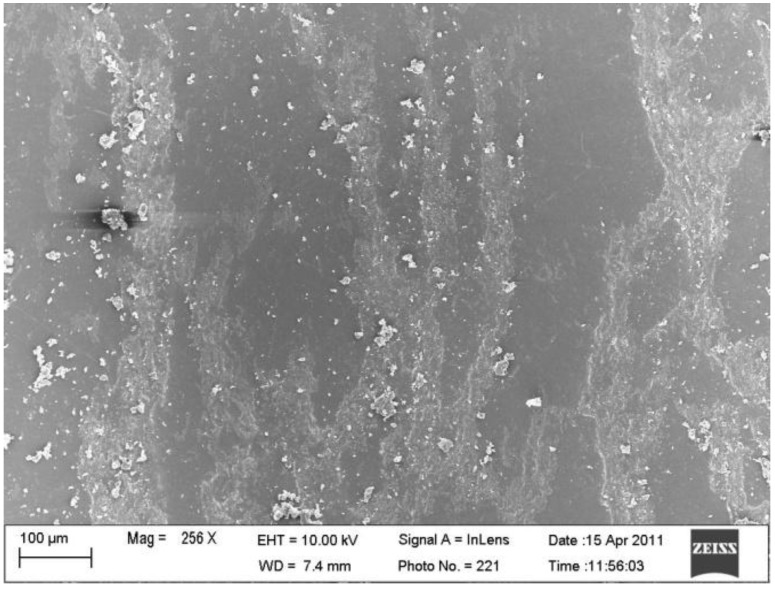
SEM image of the orientation of the nickel nanostrands observed via the thickness of the SMP nanocomposite. Reproduced with permission from [41]. Copyright 2011 RSC Publishing.

In the fabrication of an SMP nanocomposite, nickel nanostrands (or nickel coated CNTs) with different weight fractions were blended into the SMP resin and, then, vertically aligned under a magnetic field. Due to the nano-size effect, the viscosity of the mixture was increased, due to the blended nano-particles. The resulting mixture was degasified in a vacuum oven to completely eliminate air bubbles. Afterwards, the resin transfer molding process was used to fabricate SMP nanocomposites. Figure 6 reveals the morphology of the aligned nickel nanostrands [41]. The electrical properties of the SMP nanocomposites were also characterized by the four-point probe method. The measured electrical resistivity of the SMP nanocomposites embedded with different weight fractions of magnetic nanostrand alignments in the SMP matrix is obtained. Experimental results reveal that the electrical resistivity of the corresponding SMP nanocomposite decreases from 1.81 Ω cm to 0.86 Ω cm, with the increase in the weight fraction of aligned nickel nanostrands from zero to 8 wt %. Furthermore, the randomly dispersed nickel nanostrands cause the electrical resistivity of the SMP nanocomposites to decrease slightly. However, the same amount of nickel nanostrands, if well aligned upon applying a proper magnetic field, can significantly reduce the electrical resistivity. It is suggested that the alignment of nickel nanostrands could serve as conductive paths to bridge those small isolated nanostrand aggregations. Similar results are found in SMP nanocomposites incorporated with nanopaper and magnetically aligned CNTs. It is revealed that the electrical resistivity gradually decreases from 2.076 Ω·cm to 0.926 Ω·cm as the weight fraction of aligned CNTs increases from zero to 8 wt % [54].

Electrical actuation of SMP nanocomposites has been achieved with the passing of an electric current. The electrothermal mechanism has been revealed on the basis of the aligned nickel nanostrands. When an electric current passes through the nanopaper, there is electrically resistive heating occurring in the nanopaper. The resistive heating is transferred from the nanopaper to the SMP matrix resulting from the temperature difference. Here, the nickel nanostrand alignment facilitates heat transfer from the nanopaper to the underlying SMP part. The flat SMP nanocomposite incorporated with 8 wt % of vertically aligned nickel nanostrands is further utilized to demonstrate the electrically triggered actuation. It is initially bent into a “U”-like shape above the transition temperature. The deformed nanocomposite specimen begins to return to its original shape after an electrical current is applied for 10 s. The specimen shows a very low recovery ratio in the first 20 s. Faster shape recovery is followed, until 60 s. During the last 15 s, shape recovery is only slight. In the end, the SMP composite achieves a nearly 100% recovery ratio. This experiment demonstrates the contribution of the magnetic alignment in comparison with randomly dispersed conductive particles. In summary, these aligned nickel nanostrands greatly help to transfer resistive heating from the nanopaper to the underlying SMP. It should be pointed out that there is almost no negative effect on the recovery ratio of the SMP nanocomposite. Therefore, both the recovery speed and recovery ratio of the SMP nanocomposite with carbon nanopaper and magnetic alignment are significantly improved.

**Figure 7:**
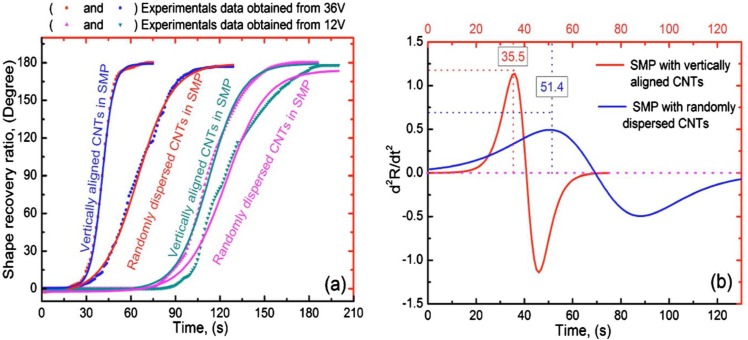
(**a**) Recovery profiles of the SMP composites with aligned and randomly dispersed 8 wt % of magnetic CNTs under 12 V and 36 V voltages, respectively; (**b**) induction and recovery times of the SMP composites for the two voltages studied. Reproduced with permission from [54].Copyright 2011 American Institute of Physics.

Moreover, in order to demonstrate the excellent recovery performance and reveal more underlying kinetics, the recovery ratio as a function of recovery time is further analyzed by introducing a standard Boltzmann function. At the beginning, the parameters in the function are calculated by the corresponding recovery ratio and recovery time. Then, the second derivative of the function is obtained. Finally, the recovery ratio and recovery time are plotted to compare the curves of the Boltzmann function. It is found that the induction time for the SMP nanocomposite with vertically aligned CNTs is 15.9 s shorter than that of the randomly dispersed CNTs under the same 36 V of triggering voltage (Figure 7 [54]).

Another strategy to fabricate a three-dimensional network is to employ a few layers of graphene (FLG) and CNFs in nanopaper. FLGs are used to significantly improve the electrical conductivity in the basal plane, while CNFs are expected to bridge the gaps among FLGs and improve the through-thickness electrical conductivity. The FLG/CNF nanopaper is then tested to see if the electrical actuation and optimization of the temperature distribution of the SMP nanocomposite has been significantly improved [55].

The electrical resistivity of the FLG/CNF nanopapers (containing a total of 1.8 g of CNFs and/or FLGs) shows a nonlinear behavior as a function of weight fraction of FLG in the nanopapers. Experimental results reveal that the volumetric resistivity gradually decreases from approximately 2.04 Ω·cm to 0.63 Ω·cm, as the weight fraction of FLGs increases from 0% to 50% in the nanopapers. This remarkable reduction in electrical resistivity is suggested to be the result of the FLGs, which serve as conductive channels to bridge those small isolated CNF aggregations in the orientation direction. When the weight fraction of FLGs increases to a critical value, the percolated network of FLGs and CNFs is formed on the entire surface, which promotes the charge transfer in the nanopaper [55]. In the CNF dominated nanopaper, the decrease in electrical resistivity becomes significant by adding in FLG, which suggests that two-dimensional FLG provides a more efficient percolating network than one-dimensional CNFs. This unconventional application of graphene, employing a synergistic effect between two-dimensional FLGs and one-dimensional CNFs, significantly improves the electrical properties of the nanopaper.

The effectiveness of the FLG/CNF nanopaper on the electrical actuation of an SMP nanocomposite is demonstrated. The tested SMP nanocomposite (with dimensions of 60 × 10 × 1.98 mm^3^) was prepared into a “Π” shaped geometry. The electrically induced shape recovery was slow within the first 9 s. After that, the recovery speed of the SMP nanocomposite specimen dramatically increased, until 72 s. At 96 s, the shape recovery of the tested nanocomposite specimen was complete and no noticeable deformation was observed after that. The recovered shape of the specimen was approximately 95% compared with its original shape. Furthermore, temperature distribution along two different conductive pathways was obtained. Experimental results reveal that higher temperature is observed where internal strain is higher during the recovery process, which is attributed to the higher local resistivity. The resting curve of the temperature distribution along the conductive pathway is relatively flat, which is the result of the homogeneous properties of the FLG/CNF nanopaper.

## 5. Conclusions and Future Perspective

In this review, we summarized the basic design and mechanisms behind the nanoscale design of nano-sized particle-enabled SMP nanocomposites for electrical actuation. A number of examples were presented to reveal the underlying mechanism and development in electrically-induced actuation of SMPs integrated with nanopaper. As we can see, a one-dimensional conductive alignment is utilized to render an SMP nanocomposite with a relatively low electrical resistivity. Consequently, two-dimensional conductive nanopaper or non-woven CNFs are utilized to simultaneously make an SMP nanocomposite with a relatively low electrical resistivity and a relatively fast electrical response. Furthermore, a three-dimensional conductive network is utilized to simultaneously make the SMP nanocomposite with a relatively low electrical resistivity and a high recovery ratio. Therefore, the advantages of such SMP nanocomposites are high electrothermal effectiveness, low energy loss, fast responsive capability, high strength, high mechanical strain energy storing ability and controllable and reversible performances, among others. With our efforts in the last six years, an improved electrical SMP nanocomposite is expected to be better prepared for more real engineering applications.
